# Pre-Procedural Right Ventricular Longitudinal Strain and Post-Procedural Tricuspid Regurgitation Predict Mortality in Patients Undergoing Transcatheter Aortic Valve Implantation (TAVI)

**DOI:** 10.3390/jcm10245877

**Published:** 2021-12-15

**Authors:** Hazem Omran, Alberto Polimeni, Verena Brandt, Volker Rudolph, Tanja K. Rudolph, Sabine Bleiziffer, Kai P. Friedrichs, Lothar Faber, Zisis Dimitriadis

**Affiliations:** 1 Clinic for General and Interventional Cardiology/Angiology, Herz- und Diabeteszentrum NRW, Ruhr-Universität Bochum, 32545 Bad Oeynhausen, Germany; verena.brandt@gmx.net (V.B.); vrudolph@hdz-nrw.de (V.R.); trudolph@hdz-nrw.de (T.K.R.); kpfriedrichs@hdz-nrw.de (K.P.F.); faber-lothar@t-online.de (L.F.); 2 Division of Cardiology, Department of Surgical and Medical Sciences, Magna Græcia University of Catanzaro, 88100 Catanzaro, Italy; polimeni@unicz.it; 3 Clinic for Thoracic and Cardiovascular Surgery, Herz- und Diabeteszentrum NRW, Ruhr-Universität Bochum, 32545 Bad Oeynhausen, Germany; sbleiziffer@hdz-nrw.de; 4 Department of Cardiology, University Hospital Frankfurt, 60590 Frankfurt, Germany; dimitriadis.zisis@gmail.com

**Keywords:** aortic stenosis, TAVI, RV function, speckle-tracking echocardiography, tricuspid regurgitation

## Abstract

Background: Right ventricular (RV) dysfunction has been linked to worse outcomes in patients undergoing TAVI. Assessment of RV function is challenging due to its complex morphology. RV longitudinal strain (LS) assessed by speckle-tracking echocardiography (STE) is a novel measure that may overcome most of the limitations of conventional echocardiographic parameters of RV function. The aim of current study was to assess the prognostic value of RV LS in patients undergoing TAVI and to assess echocardiographic predictors of long-term mortality. Methods and results: A retrospective analysis of all consecutive patients who underwent TAVI at our hospital between 1 January 2015 and 1 June 2016. Indication for TAVI was approved by a local heart-team. Echocardiographic data at baseline and after TAVI were re-analyzed and RV LS was measured in all patients with adequate image quality. A total of 229 patients were included in our study (mean age 83.8 ± 5 years, 62% women, mean EuroSCORE II 5.7 ± 5%). All-cause mortality occurred in 17.3% over a mean follow-up of 929 ± 373 days. In multivariate analysis, only baseline average RV free-wall LS (HR 1.05, 95% CI (1.01 to 1.10), *p* = 0.049) and more than mild tricuspid valve regurgitation (TR) after TAVI (HR 4.39, 95% CI (2.22 to 8.70), *p* < 0.001) independently increased the risk of all-cause mortality at long- term follow-up (2.5 years), while conventional echocardiographic parameters of RV function did not predict mortality. Conclusion: Pre-procedural RV LS and post-procedural tricuspid regurgitation significantly predicted long-term all-cause mortality in patients undergoing TAVI while conventional echocardiographic parameters of RV function failed in predicting long-term outcome. RV longitudinal strain by STE should be considered in the routine echocardiographic assessments of patients with severe AS.

## 1. Introduction

Transcatheter aortic valve implantation (TAVI) is an established treatment option for patients with symptomatic sever aortic stenosis (AS) at moderate to high operative risk [1,2]. Right ventricular (RV) function impacts outcome after both surgical and transcatheter aortic valve replacement [3]. However, echocardiographic assessment of RV function is challenging because of the complex RV morphology. Conventional echocardiographic parameters like tricuspid annular plane systolic excursion (TAPSE) and fractional area change (FAC) have several limitations. TAPSE measures only the longitudinal movement of the basal lateral segment of the tricuspid annulus and FAC is largely dependent on the imaging plane. Moreover, both parameters are affected by the interrogation angle and by regional abnormalities of the RV free wall [4,5]. RV longitudinal strain (LS) analysis by speckle-tracking echocardiography (STE) is a novel method that directly measures the myocardial deformation of all segments of the RV free wall throughout the whole cardiac cycle and is less angle-dependent than conventional echocardiographic parameters [5]. RV LS has already been shown to be predictive of mortality after cardiac surgery [6]. However, there is only limited data on the prognostic value of RV LS in patients undergoing TAVI. The aim of this study was to assess the predictive value of RV function (including RV LS) on long-term all-cause mortality in patients undergoing TAVI and to look for other echocardiographic predictors of outcome after TAVI.

## 2. Methods

Patients: We performed a retrospective analysis of all consecutive patients treated at our hospital with TAVI due to symptomatic severe aortic stenosis (AS) between 1 January 2015 and 1 June 2016. Study inclusion criteria were: severe degenerative AS as determined by echocardiography with a valve area ≤1.0cm^2^ (or <0.60 cm^2^/m^2^ of body surface area; BSA) and a high-gradient (mean pressure gradient (PG) ≥ 40 mmHg in patients with normal LV-function and normal flow status with stroke volume index > 35 mL/m^2^) or low-flow low-gradient AS (in patients with LVEF < 50% with stroke volume index < 35 mL/m^2^). Patients should be symptomatic (primarily dyspnea on exertion of at least New York Heart Association NYHA II) or having other symptoms like angina pectoris or syncope on exertion due to the severe AS. All patients should have a high operative risk for conventional aortic valve replacement with a logistic EuroSCORE of ≥20% and an endorsement from local heart-team to perform TAVI. We excluded patients in cardiogenic shock or with recent myocardial infarction (<2 weeks) as well as patients who did not have sufficient echocardiographic data available to assess the RV-function.

**Figure 1:**
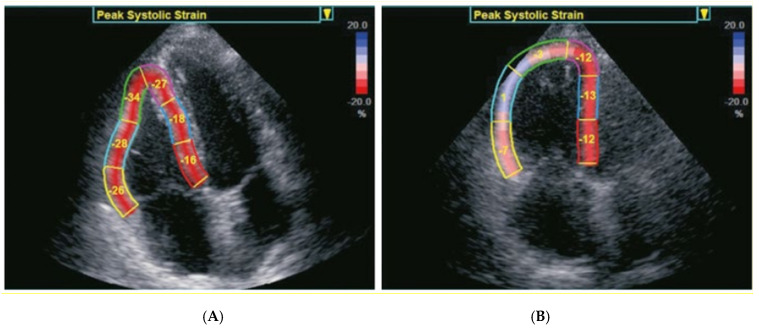
Examples of right ventricular strain analysis by speckle-tracking echocardiography. (**A**) Patient with a normal RV function. (**B**) Patient with a reduced RV function.

Transthoracic echocardiography (TTE): Each patient underwent standardized 2D TTE for the determination of RV dimensions and functional parameters according to the recommendations of the American Society of Echocardiography using commercially available ultrasound scanners (GE Healthcare Vivid E 90, Waukesha, WI, USA). Echocardiographic views, including apical four- and two-chamber views (A4Ch, A2Ch) as well as parasternal long- and short-axis views, in the left lateral decubitus position, were obtained in 2D, color Doppler, and color tissue Doppler imaging (TDI) modes. The 2D images were acquired with the frame rate of at least 40 FPS. The images with sufficient image quality were selected for postprocessing analysis. Special care was taken to fully visualize the whole of the RV with the RV focused A4Ch views. Tricuspid annular plane systolic excursion (TAPSE) was measured in A4Ch by using M-mode or anatomical M-mode, if necessary, defined as the maximal excursion at the most lateral aspect of the tricuspid annulus. RV basal free wall velocities (S’) were obtained from TDI on the lateral TV annulus in A4Ch. RV fraction area change was analyzed from A4Ch views. The RV endocardium was traced at end-diastole (end-diastolic area: EDA) and at end-systole (end-systolic area: ESA), and FAC was calculated as (EDA-ESA/EDA) multiplied by 100. Right ventricular systolic pressure (RVSP) was estimated by measuring the peak systolic tricuspid regurgitant velocity flow with continuous-wave Doppler in patients with tricuspid valve regurgitation. Myocardial deformation derived from speckle-tracking echocardiography (STE) analyses was performed according to the recommendations of the American Society of Echocardiography and European Association of Cardiovascular Imaging. Analyses were performed based on a right ventricle-focused view. Tricuspid annulus was marked with two points (lateral and septal), and a third point was placed at the RV apex. The endocardial border was automatically tracked throughout the cardiac cycle. Manual corrections were performed to optimize region of interest position as necessary. Longitudinal strain measurements were derived from STE analysis throughout the whole cardiac cycle using the available software (EchoPac Version 204), and the peak LS was recorded. Figure 1 showes exapmles of RV LS analyses by STE. Echocardiographic assessments were performed at baseline and after the TAVI procedure before discharge.

Patients were followed up after discharge through routine phone calls and standardized questionnaires. Data on mortality were collected from local registry offices and by contacting patients’ general practitioners. Patients signed informed consent forms before the procedure that allowed collection of data and future contacts (phone calls and mail) and participation in our local registry. The study was approved by the local ethics committee and was performed in accordance with the Declaration of Helsinki and the Guidelines for Good Clinical Practice.

## 3. Statistics

Statistical analyses were performed using IBM SPSS V22 (IBM Corporation, Armonk, NY, USA) and the MedCalc Statistical Software version 14.8.1 (MedCalc Software, Ostend, Belgium). Data was expressed as percentages for categorical variables and as mean ± standard deviation (SD) or median ± interquartile range (ICR) for continuous variables. Continuous variables were compared using Students t and Mann–Whitney U tests as appropriate. Differences between multiple groups with a normal distribution were compared by one-way ANOVA. Within-group differences were analyzed using repeated measures ANOVA or paired t-test. If no normal distribution was found, ANOVA on ranks (Kruskal–Wallis) was performed, and Wilcoxon signed-rank test was used for within-group comparisons. Comparisons of categorical variables between groups were performed by Pearson’s X^2^ test, and for expected frequencies <5 by Fisher’s exact test.

Kaplan–Meier analysis was used to derive the event rates and plot time-to-event curves. Univariate Cox regression analysis included all echocardiographic parameters available. The variables with a *p* < 0.1 were introduced in a stepwise multivariable model and parameters with a *p*-value ≤ 0.05 were then considered statistically significant, as previously described [7].

## 4. Results

A total of 241 patients underwent TAVI at our hospital between 1 January 2015 and 1 June 2016 and 229 of them were included in our study while 12 patients have been excluded due to insufficient echocardiographic data. The mean age of included patients was 83.8 ± 5 years and 62% were women. Mean EuroSCORE II was 5.7 ± 5%. Mean baseline LVEF was 51.4 ± 13.8% and a total of 37 patients (16%) had severe low-flow low-gradient AS. Further baseline characteristics are represented in Table 1. Of note, a total of 34 patients (14.8%) had a permanent pacemaker at baseline, while 27 patients (12%) received a new permanent pacemaker after TAVI. Only one patient developed a pacemaker induced moderate TR after pacemaker implantation following TAVI.

### 4.1. Echocardiographic Assessment

The mean aortic valve area (AVA) before TAVI was 0.72 cm^2^ ± 0.16 cm^2^ with a mean pressure gradient of 47.0 ± 16.9 mmHg. Both morphologic and functional RV parameters were analyzed before and after TAVI (before discharge) and no significant differences were found except for RV apical wall strain which showed an improvement after TAVI (Table 2).

### 4.2. Echocardiographic Predictors of Long-Term Mortality

All-cause mortality occurred in 17.3% over a mean follow-up of 929 ± 373 days (Supplementary Figure S1), while in-hospital mortality was zero. Univariate cox regression analysis showed that baseline RV basal and mid-wall LS as well as average RV free wall LS were significant predictors of long-term all-cause mortality. However, traditional RV function parameters, e.g., FAC and TAPSE, were not significantly associated with all-cause mortality. Interestingly, the presence of more than mild tricuspid regurgitation at baseline (which was found in 19% of patients) was also a predictor of all-cause long-term mortality (Table 3).

Regression analysis of post-interventional echocardiographic parameters of RV function did not reveal any significant predictors of long-term all-cause mortality except for moderate to severe tricuspid regurgitation (HR 4.39, 95% CI [2.22–8.70], *p* = 0.0001).

Interestingly, in multivariate analysis, only baseline average RV free-wall LS (HR 1.05, 95% CI [1.01–1.10], *p* = 0.049) and Post-TAVI Tricuspid valve regurgitation (>mild) (HR 4.39, 95% CI [2.22–8.70], *p* < 0.001) independently increased the risk of the primary endpoint at long-term follow-up (Table 3).

## 5. Discussion

This study analyzed the association between RV function and mortality in patients undergoing TAVI. We found that RV function assessed by STE, but not by conventional echocardiographic parameters, predicted long-term all-cause mortality after TAVI. Furthermore, more than mild tricuspid regurgitation on post-procedural echocardiography was also a significant predictor of all-cause long-term mortality.

There is a growing body of evidence about the impact of RV dysfunction on mortality after TAVI [3,8,9]. However, conventional echocardiographic parameters for assessment of RV function have demonstrated conflicting results with respect to their association with mortality after TAVI [10,11,12]. The advantage of RV free-wall LS assessed by STE over conventional echocardiographic parameters has been demonstrated by Scheuler et al. [13] and by Medvedovsky et al. [14] reporting a significant association with mortality after TAVI. Our study results are in line with their results and confirm the better performance of strain analysis by STE and its prognostic value in patients undergoing TAVI. The advantages of strain analysis by STE over conventional echocardiographic parameters can be linked to many reasons. First, RV strain analysis includes the entire RV free wall, in contrast to TAPSE and S’, which assess only the basal segment of the RV. Second, RV strain analysis tracks the myocardium over the whole cardiac cycle which allows for the detection of the maximal and minimal values of deformation regardless of their cycle timing as opposed to conventional parameters like FAC, which uses only two single frames at end- diastole and end-systole. Third, STE imaging does not depend much on the ultrasound interrogation angle as TAPSE and S’ do. Therefore, the assessment of RV strain by STE should be increasingly adopted in the everyday practice.

Regarding tricuspid regurgitation (TR) we found a significant association between >mild TR at baseline and after TAVI with long-term all-cause mortality in the univariate analysis. However, the multivariate analysis showed that only post-procedural >mild TR to be significantly predictive of mortality. The significance of baseline TR is not well established as it is affected by the postcapillary pulmonary hypertension caused by the severe aortic stenosis. Moreover, the degree of TR could significantly change after TAVI due to improvement of the hemodynamics at the left sided heart. As such there are still conflicting data on the prognostic value of baseline TR in patients undergoing TAVI [3,12,15]. However, we found that persistence of more than mild TR after TAVI to be predictive of worse long-term outcomes, which strengthens previous study results [3,15]. It has been already shown that the degree of LV hypertrophy might decrease following aortic valve replacement, but the interstitial fibrosis of LV myocardium persists, contributing to persistent diastolic dysfunction and sustained secondary pulmonary hypertension which in turn perpetuates dilatation of the RV and worsening of TR [15,16]. On the other side, increased stroke volume after TAVI increases venous return which might be not fully accommodated by the right heart especially in an already dilated RV with a reduced function. This could also contribute to further RV dilatation and worsening of TR [15,16]. The presence of moderate to severe TR after TAVI is an independent predictor of worse outcome and warrants further assessment of the TR to identify patients who might be candidates for interventional therapy. However, the effect of interventional TR therapy on long-term outcome in patients after TAVI is a matter of further research.

## 6. Limitations

Our study has several limitations. First, due to the retrospective nature of this single-center study, results might be biased, even after adjustments in regression analyses and causal associations cannot be made. However, our hospital has a TAVI-population comparable with that of other high-volume TAVI centers, and our results are likely to be relevant to the overall TAVI community. Patients with inadequate image quality were excluded from analysis, which limits the generalizability of our results, as patients with morbid obesity or advanced lung emphysema might be especially underrepresented in our study. Follow-up echocardiography was only performed after TAVI before hospital discharge and no long-term echocardiographic assessments were available, so changes in RV function over long-term could not be assessed.

In conclusion, our study confirms the feasibility and prognostic value of baseline RV LS in patients undergoing TAVI. RV LS by STE should be considered in the routine echocardiographic assessments of patients with severe AS. Moreover, our study underlines the prognostic value of moderate to severe TR following TAVI which warrants further assessment.

## Figures and Tables

**Table 1:** Baseline characteristics.

Age (years)	83.8 ± 5
Male (n, %)	87 (38)
Weight (kg)	72 ± 14.4
Height (cm)	166 ± 8.5
COPD (n, %)	36 (15.7)
Diabetes (n, %)	48 (21)
NYHA Class II (n, %)	36 (15.7)
NYHA Class III (n, %)	177 (77.3)
NYHA Class IV (n, %)	16 (7)
Hypertension (n, %)	180 (78.6)
Coronary artery disease (n, %)	124 (54.1%)
Chronic kidney disease (n, %)	82 (35.8)
Atrial Fibrillation (n, %)	80 (34.9)
Previous Surgery (n, %)	41 (17.9)
Logistic Euroscore II	5.7 ± 5.0
STS score	5.6 ± 3.6

**Table 2:** Comparison of Pre- and Post- TAVI echocardiographic parameters.

Parameter	Pre−TAVI	Post−TAVI	*p*-Value
RV basal wall LS (%)	−21.5 ± 7.9	−20.8 ± 8.3	0.25
RV basal time to peak strain (ms)	376.4 ± 76	379.4 ± 94	0.71
RV middle wall LS (%)	−21.5 ± 8.2	−21.1 ± 8.3	0.52
RV middle wall time to peak strain (ms)	368.6 ± 72	371.8 ± 89	0.65
RV apical wall LS (%)	−15.7 ± 8.4	−17.1 ± 7.9	0.04
RV apical wall time to peak strain (ms)	407.9 ± 119.7	405 ± 112.6	0.8
Average RV free−wall LS (%)	−20.0 ± 7.6	−19.8 ± 7.8	0.7
RA volume (mL)	43.8 ± 30.3	41.6 ± 25.5	0.08
RV end−diastolic basal diameter (mm)	36.3 ± 6.6	36.7 ± 6.5	0.32
RV EDA (mm^2^)	14.6 ± 3.9	14.7 ± 4.1	0.6
RV ESA (mm^2^)	8.8 ± 3.2	8.9 ± 3.4	0.7
FAC (%)	40 ± 12.2	40.2 ± 12.4	0.8
TAPSE (mm)	16.6 ± 4.1	16.4 ± 4.2	0.28

Table legend: RV: right ventricular, LS: longitudinal strain, EDA: end-diastolic area, ESA: end-systolic area, FAC: fractional area change, TAPSE: tricuspid annular plane systolic excursion.

**Table 3:** Echocardiographic Predictors of Long-Term All-Cause Mortality.

Long-Term All-Cause Mortality				
Variable	Univariate		Multivariate	
	Hazard Ratio (95% CI)	*p*-Value	Hazard Ratio (95% CI)	*p*-Value
Average baseline RV free-wall LS	1.05 (1.01–1.10)	0.044	1.05 (1.01–1.10)	0.049
Pre-TAVI TR2 (>mild)	2.95 (1.46–5.97)	0.003	1.31 (0.55–3.15)	0.53
Post-TAVI TR (>mild)	4.39 (2.22–8.70)	<0.0001	3.77 (1.62–8.75)	0.002
TAPSE	1.0 (0.98–1.18)	0.89		
FAC	0.98 (0.96–1.01)	0.12		

Table legend: RV: right ventricular, LS: longitudinal strain, TAVI: transcatheter aortic valve implantation, TR: tricuspid regurgitation. FAC: fractional area change, TAPSE: tricuspid annular plane systolic excursion.

## Data Availability

The data underlying this article will be shared on reasonable request to the corresponding author.

## Supplementary Materials

The following are available online at https://www.mdpi.com/article/10.3390/jcm10245877/s1, Figure S1: Long-term all-cause mortality after TAVI.
