# Supplementary figures and images for: Pre-Procedural Right Ventricular Longitudinal Strain and Post-Procedural Tricuspid Regurgitation Predict Mortality in Patients Undergoing Transcatheter Aortic Valve Implantation (TAVI)
Source: J Clin Med. 2021 Dec 15;10(24):5877. doi: 10.3390/jcm10245877 (PMC8707506; doi:10.3390/jcm10245877)

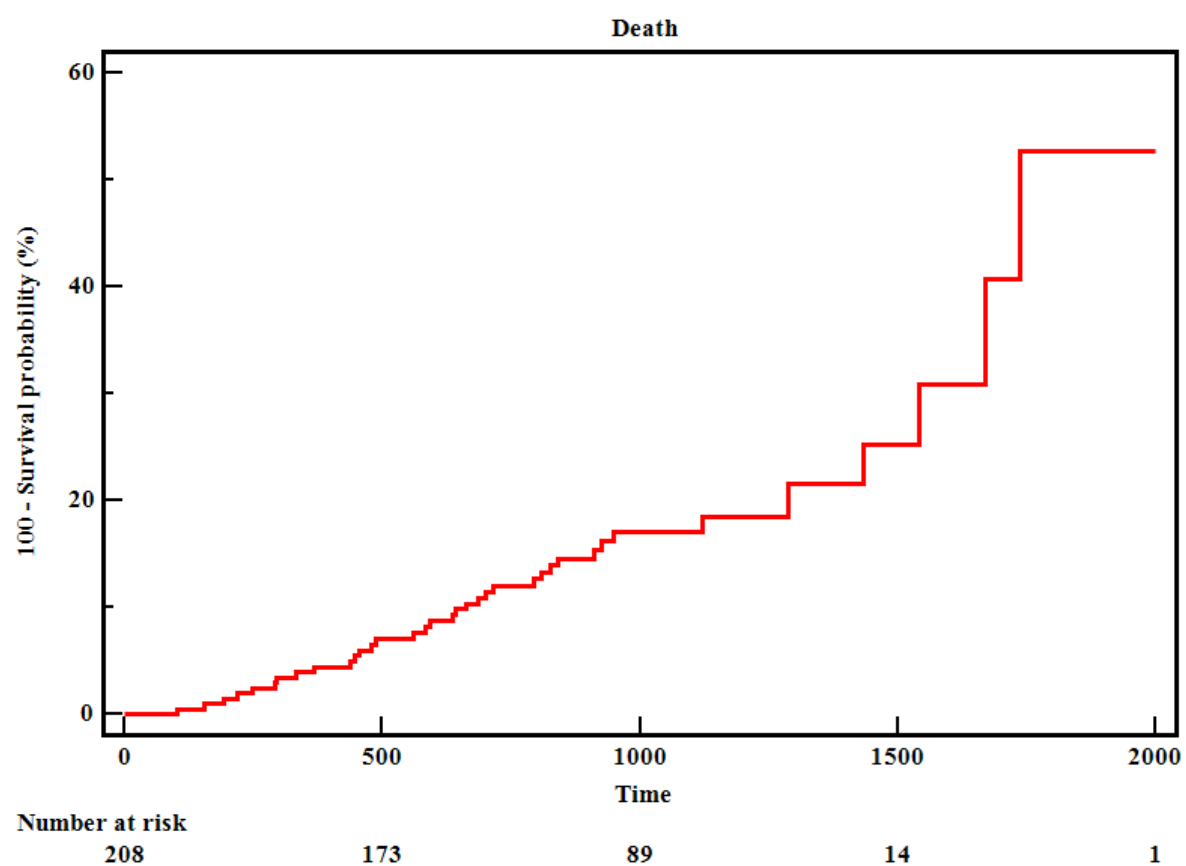

Figure S1. Long-term all-cause mortality after TAVI.

Supplement: Supplementary file 1 [file jcm-10-05877-s001.zip › jcm-1503324-supplementary.pdf]
